# Solar-active biogenic CQD-TiO_2_ nanorods: toward safe and efficient water treatment systems

**DOI:** 10.1007/s10653-026-03032-y

**Published:** 2026-02-07

**Authors:** Melek Koç Keşir, Elif Ayazoglu Demir, Mahmut Deniz Yılmaz

**Affiliations:** 1 Department of Chemistry, Faculty of Science, Karadeniz Technical University, 61080 Trabzon, Turkey; 2 Department of Chemistry and Chemical Processing Technologies, Maçka Vocational School, Karadeniz Technical University, Trabzon, Turkey; 3 Department of Basic Sciences, Faculty of Engineering-Architecture, Necmettin Erbakan University, 42090 Konya, Turkey

**Keywords:** Carbon quantum dots, TiO_2_ nanorods, Photocatalysis, Wastewater treatment, Antimicrobial activity, Cytotoxicity, Hybrid material

## Abstract

Biomass-derived carbon quantum dots (CQDs) were integrated with TiO_2_ nanorods to enhance visible-light photocatalytic performance. Photocatalytic performance was evaluated for the degradation of an organic dye and removal of a heavy metal under visible-light irradiation; the incorporation of C-CQDs reduced the band gap of TiO_2_ nanorods from 3.11 eV to 2.84 eV, resulting in enhanced photocatalytic performance with 88.04% dye degradation and 88.39% Cr(VI) reduction. In addition, the hybrid photocatalyst showed enhanced antimicrobial activity against *Escherichia coli* compared with pristine TiO_2_, while cytotoxicity assays confirmed acceptable biocompatibility. The C-CQDs/TNRs photocatalyst retained more than 95% of its initial activity after three consecutive reuse cycles, demonstrating excellent stability and reusability. This work demonstrates a promising biomass-to-functional-nanomaterial pathway for multifunctional photocatalytic systems, offering potential for wastewater treatment and microbial disinfection applications.

## Introduction

Water pollution caused by organic dyes, toxic metal ions, and pathogenic microorganisms has become a serious environmental and public health concern. Among the various treatment strategies, semiconductor-based photocatalysis has attracted significant attention due to its potential for efficient and sustainable wastewater remediation under light irradiation (Meena et al., 2025a; Xu et al., 2025a).

Titanium dioxide (TiO_2_) is one of the most widely studied photocatalysts owing to its chemical stability, non-toxicity, and strong oxidative capability (Xu et al., 2024; Zhang et al., 2021a). This approach offers a promising alternative for the photocatalytic removal of pollutants from water resources and has the potential to outperform conventional treatment methods in certain aspects (Silva et al., 2024). However, its wide band gap (3.2 eV) and rapid recombination of photogenerated charge carriers significantly limit its efficiency under visible-light illumination (Farjadfar et al., 2022; Xu et al., 2025a). Consequently, challenges such as poor visible-light absorption, reduced charge separation, and energy loss due to electron–hole recombination persist (Karaca et al., 2023). To overcome these limitations, various modification strategies have been proposed, including nanostructure engineering, coupling with other semiconductors, chemical composition tuning, dye sensitization, surface modification, and metal ion deposition and surface hybridization with carbon-based materials (Karaca et al., 2023; Meena et al., 2025b). In particular, carbon quantum dots (CQDs) have emerged as promising modifiers due to their tunable optical properties, strong light-harvesting ability, and capability to promote charge transfer at semiconductor interfaces.

Carbon quantum dots (CQDs) are novel zero-dimensional carbon-based nanoparticles with sizes below 10 nm and spherical or quasi-spherical morphology. They have attracted attention for applications in bio-imaging, photodynamic therapy, drug delivery, and optoelectronics due to their unique optical and electronic properties (Kuo et al., 2024; Prabhakaran et al., 2025; Sendão et al., 2024). Hybridization of TiO_2_ with CQDs has emerged as an effective approach to overcome TiO_2_ limitations in photocatalysis (Silva et al., 2024). The *sp*^2^-hybridized carbon framework of CQDs, interconnected via *sp*^3^-like junctions, allows them to act as electron donors and acceptors, improving charge carrier mobility and enhancing the separation of photogenerated electrons and holes in TiO_2_ catalysts (Ateş et al., 2024; Hu et al., 2022). Despite these advances, the development of environmentally benign, biomass-derived CQD/TiO_2_ hybrid photocatalysts with multifunctional performance, including pollutant degradation and antimicrobial activity, remains limited. In this context, the utilization of sustainable biomass sources for CQD synthesis offers an attractive approach to improving photocatalytic efficiency while reducing environmental impact.

To date, numerous researches have reported CQDs/TiO_2_ nanomaterials, primarily concentrating on the analysis of easily removable organic dyes, inorganic substances, and pathogens. Hu et al. demonstrated that CQDs can expand the specific surface area of TiO_2_, enhancing adsorption, while also improving visible-light absorption and electron transfer during methylene blue (MB) degradation (Hu et al., 2022). Zhang et al. (2019) highlighted that Ti–O–C bonds between CQDs and TiO_2_ facilitate efficient electron–hole separation. Smrithi et al. (2024) employed bio-based CQDs derived from Mentha piperita on TiO_2_, reducing charge recombination. Immanuvel et al. (2024) reported enhanced photocatalytic degradation of brilliant green over CQDs/TiO_2_ composites prepared from orange peel–derived CQDs. Wang et al. synthesized CQDs/TiO_2_ composites with a reduced bandgap (2.32 eV), achieving 70.63% Cr(VI) reduction under visible light (Wang et al., 2024). Nitrogen-doped CQDs (N-CQDs) combined with TiO_2_ exhibited 10.7 times higher photocatalytic activity than pure TiO_2_ due to improved electron transfer (Wei et al., 2023). Chang et al. (2022) reported a four-fold increase in Cr(VI) reduction efficiency using N, S-doped CQDs/TiO_2_ composites. CQDs/TiO_2_ composites have also been effective for a variety of pollutants including methyl orange, CO_2_, malachite green, rhodamine B, ibuprofen, congo red, bisphenol A, Cr(VI), ciprofloxacin, sulfadiazine, sulfamethoxazole, trimethoprim, maltitol, caffeine, carbamazepine, propranolol, and atenolol (Ahmad et al., 2024; Ateş et al., 2024; Guo et al., 2020; Hu et al., 2022; Meena et al., 2025a; Muangmora et al., 2023; Ponkshe & Thakur, 2022; Silva et al., 2023; Xu et al., 2023, 2024; Zhang et al., 2021b; Zhou et al., 2025). Moreover, CQDs/TiO_2_ composites show promise in antimicrobial applications. For example, Kuo et al. (2024) reported nearly 100% antibacterial efficiency against Staphylococcus aureus under UV exposure. Similarly, CQDs nanohybrids achieved photo-disinfection of *E. coli* and *S. aureus* through reactive oxygen species generation (Muangmora et al., 2025; Qu et al., 2022). Carbon quantum dot-decorated Pd/Ti^3+^-TiO_2_ nanocomposites have been explored for plant pathogen inactivation, including *Fusarium* and *Colletotrichum* species (He et al., 2022; Malmir et al., 2020; Widiyandari et al., 2023; Zhang et al., 2021c; Zhu et al., 2021). Unlike previously reported biomass-derived CQDs, the present study employs *Carduus *spp*.* as a unique precursor, enabling the formation of CQDs with distinct surface functionalities and electronic properties. These features allow the development of a multifunctional CQDs/TiO_2_ hybrid system capable of simultaneous photocatalytic degradation, photo-reduction, and biological activity assessment within a single framework.

Although numerous studies have reported CQDs/TiO_2_-based photocatalysts for the removal of organic dyes, heavy metals, or microorganisms, most of them rely on hydrothermally or chemically synthesized carbon dots and focus on a single application without evaluating material stability or biocompatibility. In addition, the use of fixed-bed pyrolysis for producing biomass-derived CQDs and their integration with one-dimensional TiO_2_ nanorods remains scarcely explored. Furthermore, comprehensive studies simultaneously addressing photocatalytic degradation, microbial inactivation, reusability, and cytotoxicity are still limited. In this study, we address these gaps by fabricating a novel C-CQDs/TiO_2_ nanorod hybrid using *Carduus *spp.-derived carbon quantum dots synthesized via a fixed-bed pyrolysis reactor. The proposed material demonstrates enhanced photocatalytic performance toward methylene blue and Cr(VI), stable reusability over multiple cycles, selective antimicrobial activity, and confirmed biocompatibility, highlighting its potential as a multifunctional and environmentally friendly photocatalyst for sustainable water treatment.

## Material and method

### Chemicals and apparatus

*Carduus *sp. is a thistle genus belonging to the *Asteraceae* family and is widely distributed in many regions. Owing to its high carbon content and abundant biomass availability, *Carduus *spp. represents a suitable and sustainable precursor for the synthesis of carbon-based nanomaterials*. Carduus *spp. samples were picked up from given coordinates (40° 54′ 26″ N, 39° 41′ 22″ E) in Trabzon, Turkey. The complexing agent 1,5-Diphenyl Carbazide (C_13_H_14_ON_4_) was obtained from LaboChemie, while commercial titanium dioxide (TiO_2_) nanoparticles in the anatase phase (≥ 99%), methylene blue, acetone, and sodium hydroxide (NaOH,  ≥ 99%) were sourced from Sigma Aldrich. Hydrochloric acid (HCl, 37%) was purchased from Carlo Erba Company, and potassium dichromate (K_2_Cr_2_O_7_,  ≥ 99.9%) was supplied by AppliChem (Panreac ITW Companies). A dual-lamp 6W irradiation source with emissions at 254/365 nm (light intensity: 350 mW/cm^2^) was secured from Spectroline ENF260. Müeller Hinton Agar (MHB) and Sabaroud Dextrose Agar (SDA) were provided by Merck Millipore. All reagents used were of analytical grade. Phosphate-buffered saline (PBS) came from Pan Biotech, and a Serva-brand dialysis bag was used for purifying C-CQDs. The microbial strains included *Escherichia coli* (ATCC 25922) and *Aspergillus niger* isolates, which were obtained from the Karadeniz Technical University's Department of Medical Microbiology and Hacettepe University's Mycology Laboratory, respectively. Deionized water for solution preparation as well as photocatalytic experiments was produced using a Merck Millipore Direct-Q® 8 UV system. Ultrasonic treatments were conducted with a Bandelin Sonorex Digitec ultrasonic bath. All chemicals were of analytical grade and used as received without additional purification. Freshly distilled water (DW) was consistently employed throughout experimentation, including for testing, treatment, and rinsing processes. Dulbecco's phosphate-buffered saline (DPBS) was also sourced from Pan Biotech. Additional reagents included 3-(4,5-dimethylthiazol-2-yl)-2,5-diphenyl tetrazolium bromide (MTT dye) and trypan blue solution, both acquired from Sigma (St. Louis, MO, USA). Other materials such as Dulbecco's Modified Eagle Medium (DMEM), fetal bovine serum (FBS), and penicillin/streptomycin were supplied by Thermo Scientific, while Trypsin–EDTA was obtained from Wisent.

### Characterization

A comprehensive set of characterization techniques was employed in the experimental analysis, including TGA, XRD, UV–Vis absorbance spectroscopy, XPS, FE-SEM/EDS, HRTEM, FTIR, fluorescence spectroscopy, and UV–Vis DRS methods. Fourier transform infrared (FTIR) spectroscopy was employed to identify the surface functional groups and chemical bonding states of the synthesized materials. The spectra were recorded in the range of 400–4000 cm^−1^ using a Perkin Elmer 1600 FTIR spectrometer at room temperature. For clarity, the spectra are presented between 500 and 4000 cm^−1^. Shimadzu UV 3600 Plus UV–Vis-NIR spectroscopy was used to investigate the optical absorption properties of the synthesized samples, using BaSO_4_ as the reflectance standard. The diffuse reflectance spectra were recorded in the wavelength range of 200–2500 nm and converted to absorbance using the Kubelka–Munk function. X-ray diffraction (XRD, PANanalytical Empyrean) analysis was employed to investigate the crystalline structure, phase composition, and structural stability of the synthesized materials. The diffraction patterns were recorded using Cu Kα radiation (λ = 1.5406 Å) operated at 60 kV and 100 mA over a 2θ range of 10°–80° at room temperature. X-ray photoelectron spectroscopy (XPS) was utilized to examine the surface elemental composition and chemical states of the synthesized samples. X-ray photoelectron spectroscopy (XPS) was conducted using a Specs-Flex XPS with monochromatic Al Kα radiation to examine the elemental composition and chemical structure of both C-CQDs/TNRs and pristine TNRs.

Photoluminescence (PL) spectroscopy was employed to investigate the charge carrier recombination behavior of the synthesized samples. The PL spectra were recorded at room temperature using an appropriate excitation wavelength. Fluorescence spectra of C-CQDs liquid samples were recorded using a Photon Technologies International Quanta Master Spectrofluorimeter (model QM-4/2006) with a slit width of 5 nm, photomultiplier tube voltage set at 400 V, and a scan speed of 1200 nm/min at room temperature. For morphological analysis and particle size distribution of the dot samples and composites, HRTEM images were collected using an FEI TALOS F200S TEM operated at 200 kV. Additionally, Field-emission scanning electron microscopy (FESEM) was employed to examine the surface morphology and microstructural features of the synthesized samples. Energy-dispersive X-ray spectroscopy (EDS), coupled with FESEM, was used to analyze the elemental composition and distribution of the constituent elements. FE-SEM and EDS analyses were performed using an FEI Quanta 650 field-emission SEM with an accelerating voltage range of 100 V–30 kV and a resolution of 1.2 nm @ 30 kV (SED)/0.8 nm @ 30 kV (STEM). EDS images were obtained via an Inca 250 SSD XMax20 detector featuring a Peltier-cooled configuration with a 20 mm^2^ active area and a resolution of 129 eV for elemental mapping. Thermogravimetric analysis (TGA) and derivative thermogravimetry (DTG) were performed to evaluate the thermal stability and decomposition behavior of the synthesized samples. Thermogravimetric analysis (TGA) of *Carduus *spp. samples was performed using an Exstar SII TG/DTA 3600 instrument in a nitrogen atmosphere, with a heating temperature range of 25–600 °C and a controlled heating rate of 8 °C/min. A sample size of 1.61 mg with particles around 200 mesh was analyzed. The carbonization of *Carduus *spp. powders (4 g) was carried out in a fixed-bed pyrolysis reactor at 320 °C with a heating rate of 5 °C/min under a nitrogen atmosphere, ensuring a reaction time of 2 h. Finally, UV–visible absorption spectra were recorded using an Analytik Jena Specord 210 Plus spectrophotometer with quartz cuvettes (10 mm optical path length) over the spectral range of 200–600 nm and a slit width of 2 nm. These measurements were utilized to determine the optical properties of C-CQDs liquid samples and to assess the removal efficiency for MB and Cr(VI).

### Synthesis of Carduus spp. based C-CQDs and C-CQDs/TNRs nanocomposites

**Fig. 1:**
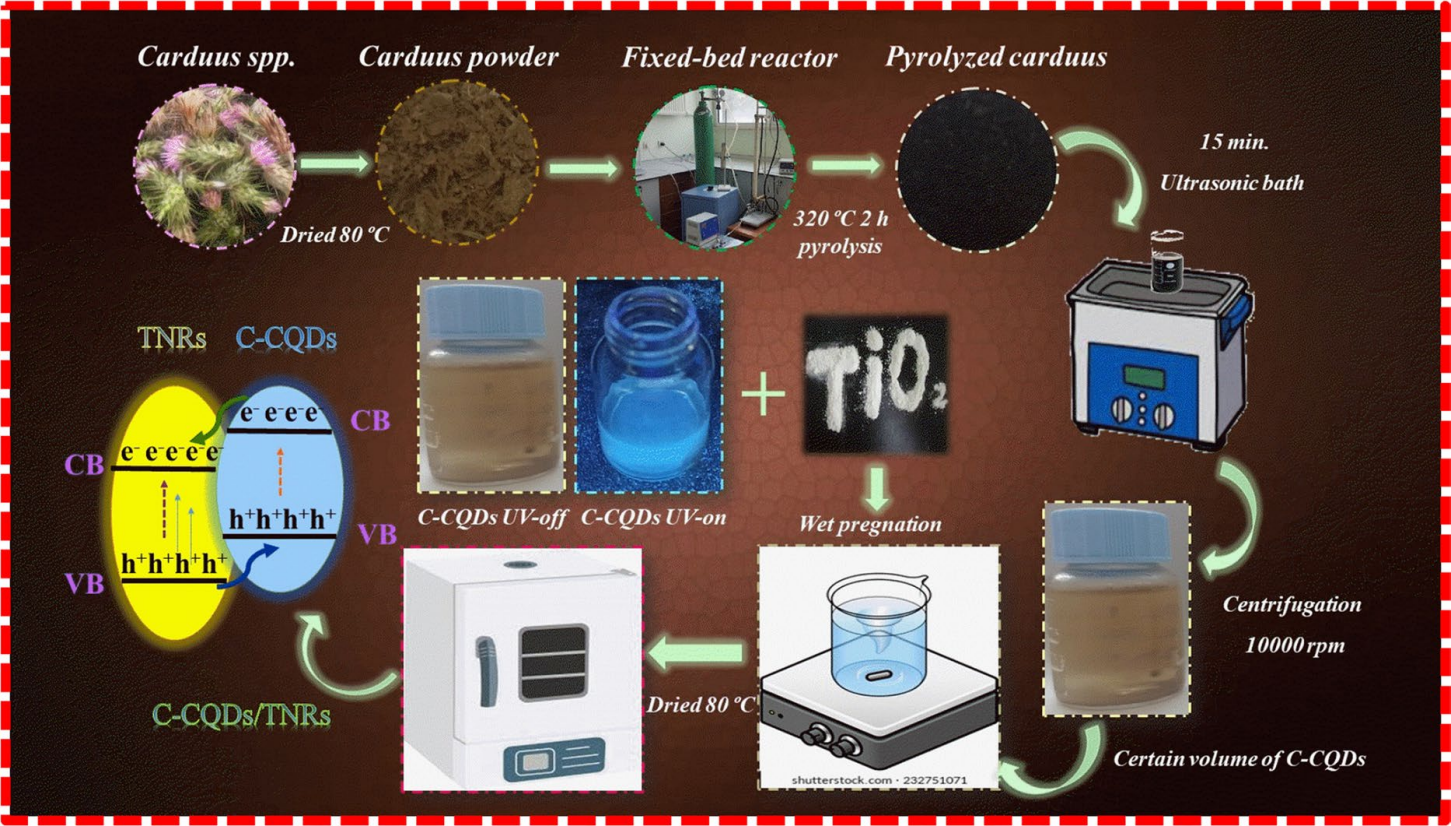
Experimental flow chart for *Carduus* spp. derived carbon quantum dots (C-CQDs) and C-CQDs/TNRs

The procedure for obtaining pyrolyzed carbon quantum dots (C-CQDs) from *Carduus *spp. and their integration onto TiO_2_ nanorods (TNRs) is depicted in Fig. 1. Initially, raw C-CQDs were produced using a vertical fixed-bed pyrolysis reactor, following the methodology described by Koç Keşir and Yılmaz (2024). In this process, approximately 4 g of finely milled *Carduus *spp. powder was loaded into the reactor, which had an internal diameter of 10 mm and a height of 700 mm, and carbonization was carried out under controlled conditions.

Essential parameters-including biomass type (~ 200 mesh), heating rate (5 °C/min), pyrolysis temperature (320 °C identified as optimal through TG/DTG analysis), carrier gas (N_2_ atmosphere) and flow rate (100 mL/min)-were carefully optimized. Carbonized *Carduus *spp. powders (0.5 g) were ultrasonically dispersed in 50 mL of deionized water for 15 min (three cycles). The resulting suspension was sequentially filtered to remove larger particles and subsequently centrifuged at 10,000 rpm for 5 min. The collected supernatant was further filtered through a 0.45 μm membrane to eliminate residual insoluble impurities. For final purification, the P-CQDs solution was dialyzed against deionized water using a regenerated cellulose dialysis membrane (MWCO: 6000–8000) for 48 h and stored at 4 °C for further analyses. Since carbon quantum dots were obtained directly as a liquid suspension, their absolute concentration could not be precisely defined; therefore, dilution ratios were expressed on a volumetric basis (v/v).

TiO_2_ nanorods (TNRs) were synthesized via a hydrothermal method. Briefly, 1 g of anatase TiO_2_ (≥ 99%) was dispersed in 80 mL of 10 M NaOH by ultrasonication, transferred to a Teflon-lined autoclave, and heated at 180 °C for 12 h. After cooling, the product was centrifuged, washed with 0.1 M HCl and distilled water until neutral pH, and finally dried at 80 °C overnight. To attach C-CQDs to the TNR surface, 0.1 g of TNRs was dispersed in 10 mL of deionized water containing different proportions of C-CQDs solution, representing 1, 5, 10, 15, 20, and 25% of the total volume. The mixtures were subjected to ultrasonic treatment for 30 min, with intermittent 15-min breaks to ensure uniform dispersion.

The resulting composites are designated as C-CQDs(X)/TNRs, where “X” denotes the percentage of C-CQDs incorporated. These one-dimensional, white-cornsilk-colored nanocomposites were collected and stored for subsequent characterization, evaluation of photocatalytic activity, and cytotoxicity assessment.

### Evaluation method of photocatalytic removal of MB and Cr (VI)

Photocatalytic experiments were carried out using 100 mg of the as-prepared C-CQDs(X)/TNRs dispersed in 100 mL of aqueous solutions containing 10 mg/L methylene blue (MB) or Cr(VI) (from potassium dichromate) in a cylindrical quartz reactor. Prior to irradiation, the suspension was stirred in the dark for 30 min to establish adsorption–desorption equilibrium. The pH of Cr(VI) solution was adjusted to ~ 1.5 using 0.1 M HCl and 0.1 M NaOH. Cr(VI) ions exist mainly as HCrO_4_^–^ at low pH, and with increasing pH they convert to CrO_4_^–^ and Cr_2_O_7_^2–^. The sorption efficiency sharply decreases from pH 1–8, with maximum removal observed at pH 1–2 due to the higher H⁺ concentration enhancing adsorption without altering the chromium speciation. Photocatalytic reactions were initiated under UV-A irradiation (350 mW/cm^2^). Aliquots (~ 5 mL) were withdrawn at regular intervals-every 15 min for MB and every 30 min for Cr(VI)-while maintaining magnetic stirring. Samples were centrifuged at 10,000 rpm for 15 min to separate the photocatalyst. Absorbance was measured at 664 nm for MB and 540 nm for Cr(VI) using a spectrophotometer. Cr(VI) concentration was quantified via the diphenylcarbazide (DPC) colorimetric method (Chang et al., 2022).

Each photocatalytic experiment was conducted in triplicate to ensure reproducibility. Removal efficiency was calculated as:

1 $$ Removal\,percentage \% = \frac{{A_{0} - A_{t} }}{{A_{0} }}*100 $$

where *A*_0_ and *A*_t_ stand for the absorbance values of the pollutants before and after a defined reaction time. After photocatalytic tests, spent C-CQDs/TNR composites were recovered by centrifugation, washed thoroughly with deionized water and absolute ethanol to remove adsorbed dyes, and dried overnight at 60 °C. For Cr(VI)-loaded composites, a preliminary soaking in 0.1 M NaOH solution was applied overnight to desorb chromium ions (Li et al., 2021; Sekar & Yadav, 2021). The materials were then rinsed multiple times with deionized water, dried, and reused in subsequent cycles to assess stability and reusability. Photocatalytic experiments were conducted separately for methylene blue and Cr(VI) using single-component aqueous solutions; no simultaneous degradation experiments were performed. All experiments were repeated triplicates, and the average values were reported; the standard deviations have been included in the graphs.

### Photokilling measurements for E-coli and A. niger

To evaluate the photocatalytic antimicrobial activity of the nanocomposites, suspensions of *Escherichia coli* and *Aspergillus niger* were prepared at concentrations ranging from 10^3^ to 10^8^ CFU/mL (equivalent to 0.5 McFarland standard). For each experiment, 1 g/L of either unmodified TiO_2_ nanorods (TNRs) or C-CQDs-functionalized TNRs were introduced into the microbial suspensions. The mixtures were exposed to a UV-A light source while continuously stirred using a magnetic stirrer for a total of 150 min. During irradiation, 10 µL aliquots were collected at time points of 0.5, 1.0, 1.5, 2.0, and 2.5 h. Each aliquot was evenly spread onto Mueller–Hinton Broth (MHB) agar plates for *E. coli* and Sabouraud Dextrose Agar (SDA) plates for *A. niger*. The inoculated plates were subsequently incubated at 37 °C for 24 h (*E. coli*) and 28 h (*A. niger*) to determine the antimicrobial efficacy of the nanocomposites.

Before irradiation, the microbial suspensions were pre-diluted to achieve appropriate cell densities: 1:5000 for *E. coli* and 1:1000 for *A. niger*. All experiments were conducted in triplicate to guarantee reproducibility, and all materials and instruments employed for the assays were sterilized by autoclaving at 121 °C for 30 min.

### Cell culture and in-vitro cytotoxicity assay

The cytotoxic potential of the synthesized C-CQDs and the hybrid C-CQDs/TNRs nanocomposite was evaluated using the MTT assay, following established protocols (Kara & Ayazoglu Demir, 2023; Saydam & Nalkıran, 2021). Two cell lines were employed: human breast adenocarcinoma cells (MCF-7, ATCC HTB-22) and human retinal pigment epithelial cells (ARPE-19, ATCC). Both cell types were maintained in Dulbecco’s Modified Eagle Medium (DMEM) supplemented with 10% (v/v) fetal bovine serum (FBS) and 1% (v/v) penicillin–streptomycin, in a humidified incubator at 37 °C with 5% CO_2_. Cells grown in T-75 flasks were detached using trypsin–EDTA, and 5000 cells per well were seeded into 96-well plates containing 200 μL of complete medium. The plates were incubated for 24 h to allow cell attachment. After this period, the culture medium was replaced with fresh medium containing various concentrations of C-CQDs and C-CQDs/TNRs, specifically 0 (control), 15.6, 31.25, 62.5, 125, 250, and 500 μg/mL. The treated cells were then incubated for an additional 48 h under the same conditions. Cell viability was determined by the MTT assay, in which metabolically active cells reduce the yellow tetrazolium salt (MTT) to an insoluble purple formazan product. Following incubation with MTT, the formazan crystals were solubilized, and absorbance was measured at 570 nm using a microplate spectrophotometer. Relative cell viability was calculated by comparing the absorbance of treated wells to that of untreated control wells, expressed as a percentage according to the following equation (Eq. 2), where OD is the optical density. This analysis provided quantitative insight into the effects of the nanocomposites on cellular survival across different concentrations.

2 $$ Cell\, Viability\,(\% ) = \frac{{OD_{Sample} - OD_{Blank} }}{{OD_{Control} - OD_{Blank} }} \times 100. $$

## Results and discussion

### Thermal stability of *Carduus spp*. powders (TG/DTG)

**Fig. 2:**
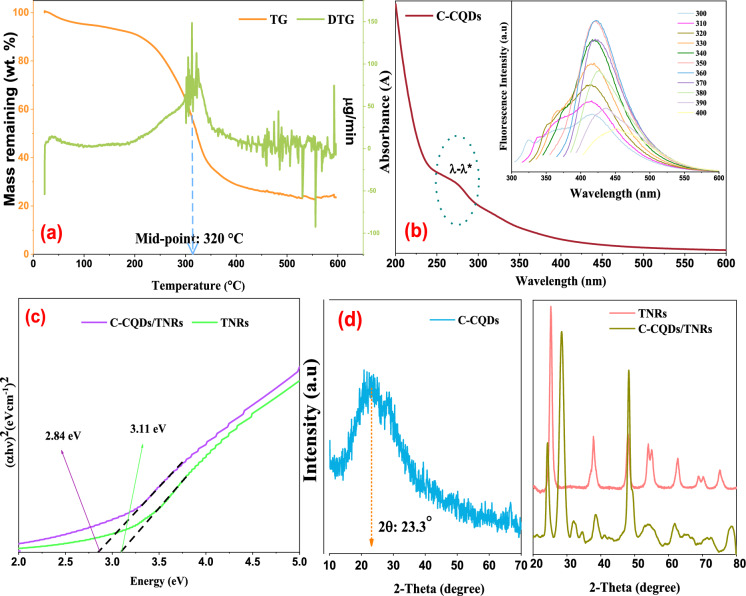
TG/DTG curve (**a**), UV–Vis spectra merged fluorescent emission spectra (**b**), Tauc plots (**c**), XRD patterns (**d**) for *Carduus* spp. derived carbon quantum dots (C-CQDs) and C-CQDs/TNRs

Thermal and chemical stability are critical parameters when evaluating the pyrolysis of biomass materials. To determine the optimal carbonization conditions for *Carduus *spp., TGA was performed. As shown in Fig. 2a, an initial weight loss of approximately 6.14% occurred between 25 °C and 120 °C, primarily due to the evaporation of adsorbed moisture during preheating. A more substantial weight reduction of 60.78% was observed between 120 °C and 345 °C, attributed to the decomposition of cellulose and hemicellulose (Tiwari & Vinu, 2025).

Lignin degradation occurred over a wider temperature range (150–900 °C) due to its amorphous structure and heterogeneous polymeric chains with varying bond energies (Escalante et al., 2022). The shoulder observed in the derivative thermogravimetric (DTG) curve at around 320 °C coincides with the region of the highest mass loss rate in the sample, as directly observed from the TG/DTG profiles. This thermal behavior guided the selection of 320 °C as the pyrolysis temperature.

### UV–vis and photoluminescence studies of C-CQDs

The optical properties of the aqueous C-CQDs solution were examined using UV–visible and fluorescence spectroscopy (Fig. 2). Prior to measurements, the solution was diluted 50-fold for UV–Vis and tenfold for photoluminescence analysis. The absorption peak at 270 nm was assigned to the π–π* transitions of aromatic C=C bonds, indicating the presence of conjugated π-electron systems (Fig. 2b). Fluorescence spectra recorded at excitation wavelengths between 300 and 400 nm revealed an excitation-dependent emission behavior (Fig. 2b), with maximum emission intensities at 423 nm and 421 nm under 350 nm and 360 nm excitation, respectively. This red-shifted emission is likely associated with surface defects introduced by functional groups on the C-CQDs (Sekar & Yadav, 2021; Zhou et al., 2025). Moreover, localized *π* and π* energy levels within *sp*^2^ domains embedded in the *sp*^3^ matrix contribute to the observed photoluminescence (Taghiloo et al., 2024).

### UV–Vis diffuse reflectance spectroscopy studies (DRS)

The optical bandgap of the samples was estimated using Tauc plots derived from the Kubelka–Munk function of the absorption spectra (Fig. 2c). The linear region of the plot was extrapolated to intersect the energy axis to determine the forbidden bandgap (Eg), according to the Eqs. (3) and (4):

3 $$ \left( {\alpha h\nu } \right)^{2} = A\left( {h\nu - E_{g} } \right) $$

4 $$ h\nu = hc/\lambda $$

in which, α represents the optical absorption coefficient; A denotes proportionality constant, c (3 × 10^8^ m s^−1^) corresponds to the speed of light, h (6.626 × 10^–34^ J Hz^−1^) is Planck's constant, and v refers to the frequency of the radiation (Chang et al., 2022).

As depicted in Fig. 2c, the band gap energy of the C-CQDs/TNRs nanocomposite decreased to 2.84 eV, compared to 3.11 eV for pure TNRs. This reduction in the forbidden band gap led to a red shift in the light absorption spectrum of C-CQDs/TNRs, thereby effectively extending the material's UV–Vis light utilization range. (Bui et al., 2025) suggested that the enhanced light absorption is likely due to the incorporation of CQDs, which induces this red shift and improves absorption in the visible region.

### XRD analysis

X-ray diffraction (XRD) was employed to analyze crystal structure, phase purity, and crystallite size of both C-CQDs and C-CQDs/TNRs over a 2θ range of 10°–80° (Fig. 2d). The broad peak at 23.3° corresponds to the (100) plane of graphitic carbon in C-CQDs (Khojiev et al., 2023). Characteristic diffraction peaks of anatase TNRs at 25.19°, 37.85°, 48.13°, 53.99°, 55.10°, 62.74°, 68.86°, 70.35°, and 75.17° were indexed to the (101), (004), (200), (105), (211), (204), (116), (220), and (215) crystal planes, respectively (JCPDS No. 21-1272). An additional peak at 27.53° was attributed to the (110) plane of rutile TNRs, suggesting that C-CQDs incorporation hinders the phase transformation of TNRs (Ateş et al., 2024; Prabhakaran et al., 2025). Integration of C-CQDs also caused distortions in the anatase lattice, reflected in both shifts in diffraction angles and changes in peak intensities. This is likely due to defect formation and particle aggregation induced by C-CQDs penetration into the TNRs matrix (Tong et al., 2022; Zhang et al., 2021d). Moreover, crystallite sizes were estimated using the Debye–Scherrer equation based on the (101) reflection, yielding average values of 13.95 nm for pristine TNRs and 9.56 nm for C-CQDs/TNRs, confirming that C-CQDs incorporation reduces the crystallite size and introduces lattice distortions.

### FTIR Studies

**Fig. 3:**
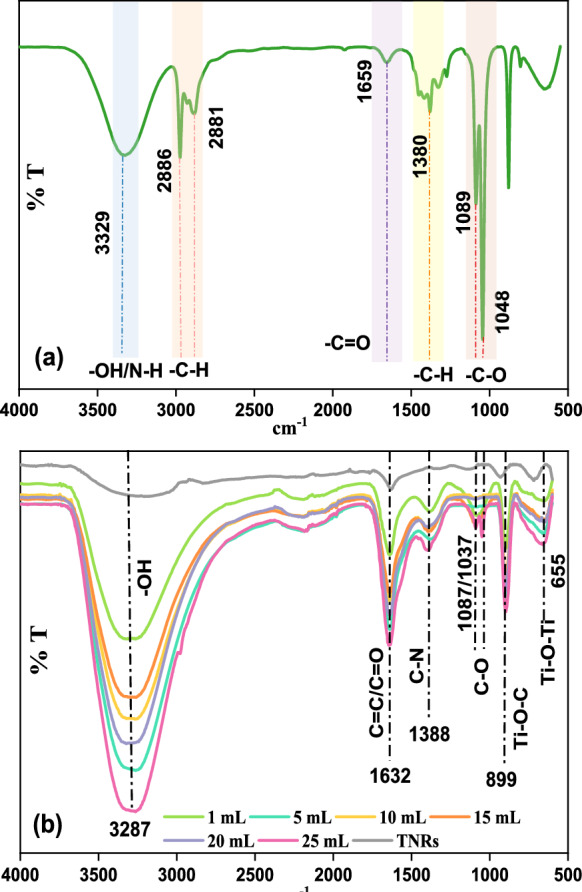
FTIR spectra of **a** as-prepared C-CQDs, **b** C-CQDs/TNRs nanocomposite

Fourier-transform infrared (FTIR) spectroscopy was employed to identify functional groups and bonding interactions in C-CQDs, TNRs, and C-CQDs/TNRs composites (Fig. 3).

In the spectrum of C-CQDs (Fig. 3a), a broad absorption band at 3323 cm^−1^corresponds to –OH and –NH stretching vibrations, reflecting the hydrophilic nature of the carbon dots. Peaks observed between 2886 and 2881 cm^−1^ are attributed to C–H stretching. The characteristic vibrations of C=O were detected around 1659 cm^−1^, while signals at 1380 and 1089–1048 cm^−1^ indicate the presence of C–H and C–O functional groups, respectively (Karaca et al., 2023; Prabhakaran et al., 2025; Xu et al., 2024). For the C-CQDs/TNRs hybrid (Fig. 3b), the broad band at 3287 cm^−1^ suggests hydrogen bonding between hydroxyl groups on the C-CQDs and TNRs surfaces (Prabhakaran et al., 2025). The peak at 1632 cm^−1^ can be assigned to C=C/C=O stretching, confirming the successful integration of carbon quantum dots into the TiO_2_ nanorods (Hu et al., 2022; Meena et al., 2025b). Additionally, new peaks appearing around 1087 cm^−1^ are indicative of C–O and C=C vibrations (Zhang et al., 2021d). A band at 899 cm^−1^ corresponds to Ti–O–C linkages formed through interactions between the carbon dots and TiO_2_. Furthermore, the C-CQDs/TNRs composite exhibits a characteristic TNRs band at 655 cm^−1^, which supports the formation of Ti–O–C and Ti–O–Ti bonds, consistent with XPS findings (Deng et al., 2021; Taghiloo et al., 2024).

### SEM–EDX and HRTEM analysis

**Fig. 4:**
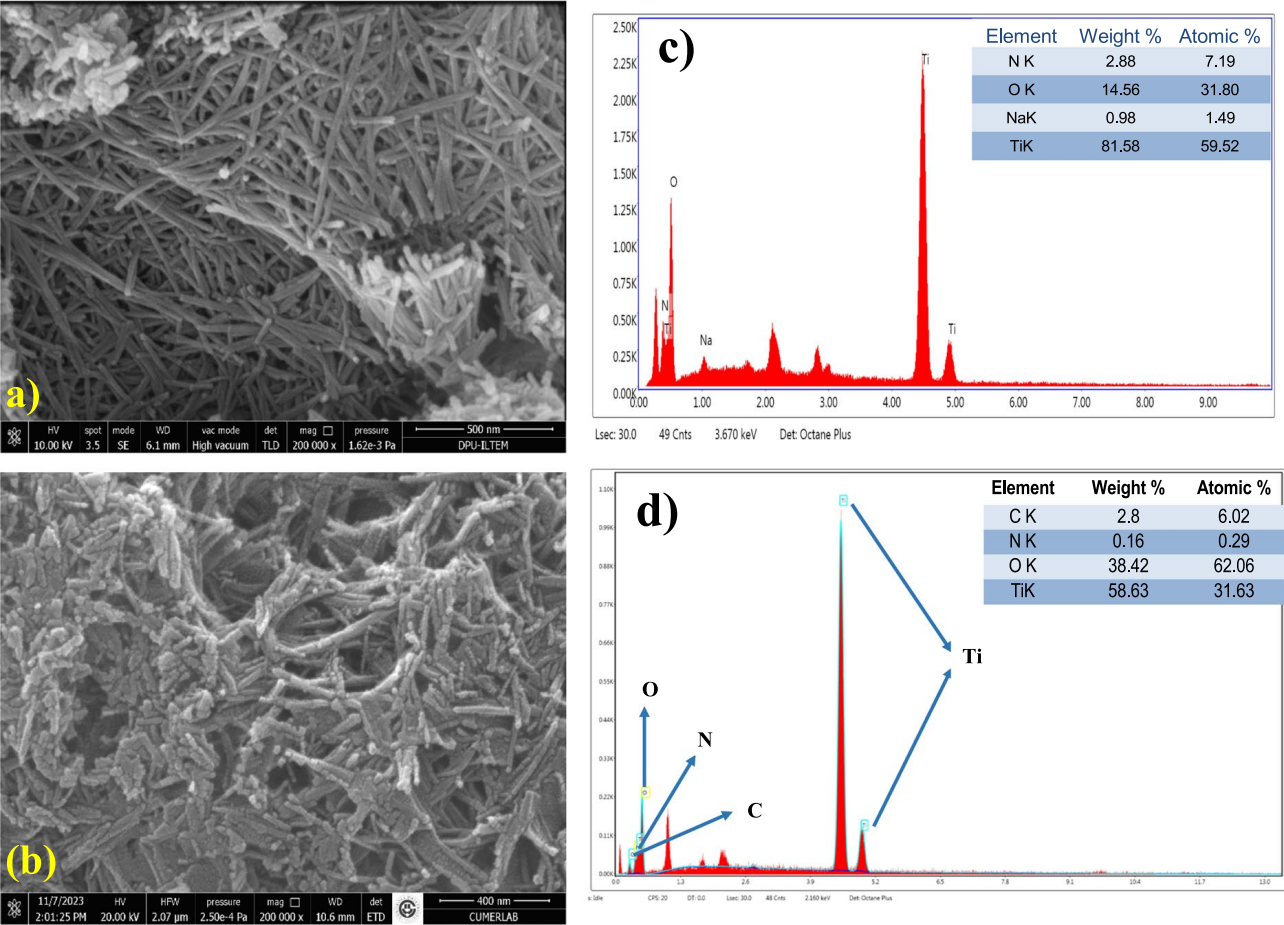
FESEM/EDS image of as-prepared TNRs (**a**–**c**), C-CQDs/TNRs nanocomposite (**b**–**d**)

Field-emission scanning electron microscopy (FESEM) images (Fig. 4) reveal that the overall rod-like morphology of TNRs remains largely unaltered upon C-CQDs incorporation. This observation confirms that integrating C-CQDs into the TNRs structure does not alter the morphology of TNRs particles, despite the dot molecules enhancing the overall optical properties (Khojiev et al., 2023).

Both pristine TNRs and C-CQDs/TNRs exhibit highly aggregated rods, making direct visualization of the carbon dots challenging. Namely, the presence of C-CQDs can hardly be perceived from the SEM images of Fig. 4. To further verify the incorporation of C-CQDs, EDS analysis was conducted to detect their presence on the TNRs surface. EDS analysis of the pristine TiO_2_ nanorods (TNRs) revealed the presence of Ti and O as the dominant elements, confirming the successful formation of TiO_2_ nanostructures. The weight and atomic percentages differed due to the significant difference in atomic masses between Ti and O, as well as the semi-quantitative and surface-sensitive nature of EDS, which is known to underestimate light elements such as oxygen. After the incorporation of C-CQDs, additional C and trace N signals were clearly detected in the EDS spectrum of C-CQDs/TNRs, indicating the successful loading of CQDs onto the nanorod surface. Compared to pristine TNRs, the relative decrease in Ti content and increase in O and C atomic percentages can be attributed to surface modification by carbon-based quantum dots rich in oxygen-containing functional groups (e.g., –OH, C=O, –COOH). These surface effects, together with the inherent limitations of EDS quantification, account for the observed variations in elemental composition. Moreover, the data suggest that carbon incorporation slightly reduces Ti content, indicating partial substitution of Ti atoms by carbon from C-CQDs (Smrithi et al., 2024; Tong et al., 2022).

**Fig. 5:**
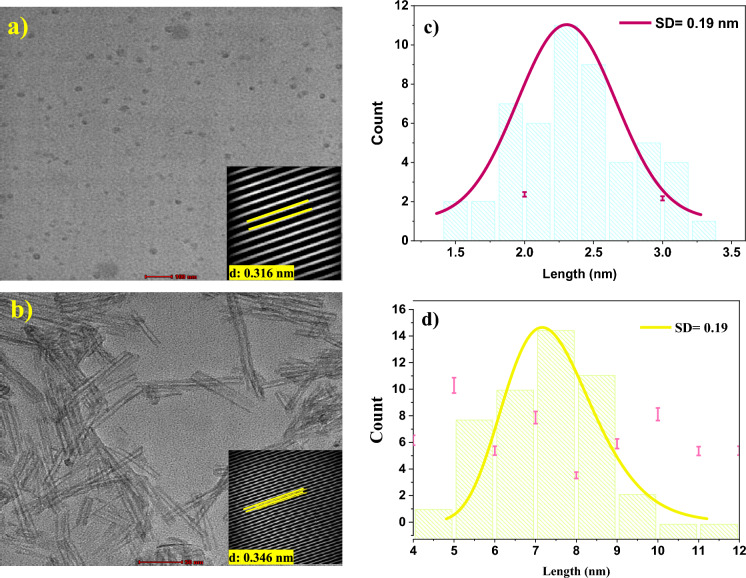
HRTEM image of as-prepared C-CQDs (**a**–**c**), C-CQDs/TNRs nanocomposite (**b**–**d**)

High-resolution transmission electron microscopy (HRTEM) further corroborated these findings. As shown in Fig. 5a–c, C-CQDs appear as quasi-spherical particles, uniformly dispersed without significant agglomeration, with diameters ranging from 1.3 to 3.5 nm and an average size of 2.38 ± 0.19 nm. The measured interlayer spacing of 0.316 nm corresponds to the (002) plane of graphene-like disordered carbon (Debnath et al., 2022; Jin et al., 2024; Nguyen et al., 2024).

In the C-CQDs/TNRs composite (Fig. 5b–d), the nanostructures exhibit a rod-like morphology with slight aggregation and an average diameter of 7.33 ± 0.19 nm. TNRs are well-dispersed within the composite, with sizes between 4 and 12 nm. Lattice fringes of 0.346 nm correspond to the (101) plane of anatase TiO_2_, confirming the preservation of crystallinity within the composite framework (Enzhou et al., 2019; Mahammed Shaheer et al., 2021; Tang et al., 2016).

### XPS analysis

**Fig. 6:**
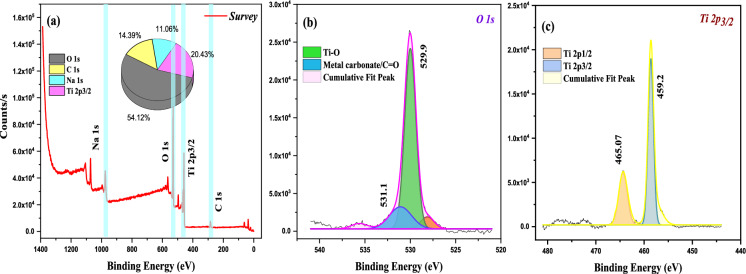
**a** XPS full scan survey spectra, deconvoluted **b** O 1* s*, **c** Ti 2*p*_3/2_ high-resolution XPS spectra of synthesized TNRs

X-ray photoelectron spectroscopy (XPS) analysis was carried out to determine the surface elemental composition and chemical states of the samples. Survey spectra were recorded to identify the constituent elements. Elemental atomic percentages were calculated from the integrated peak areas after Shirley background subtraction, applying the corresponding sensitivity factors provided by the instrument software. The survey spectrum of pristine TNRs (Fig. 6) shows characteristic peaks of C 1*s* (~ 285 eV), Ti 2*p* (~ 459 eV), and O 1*s* (~ 530 eV), confirming the formation of TiO_2_ nanorods.

**Fig. 7:**
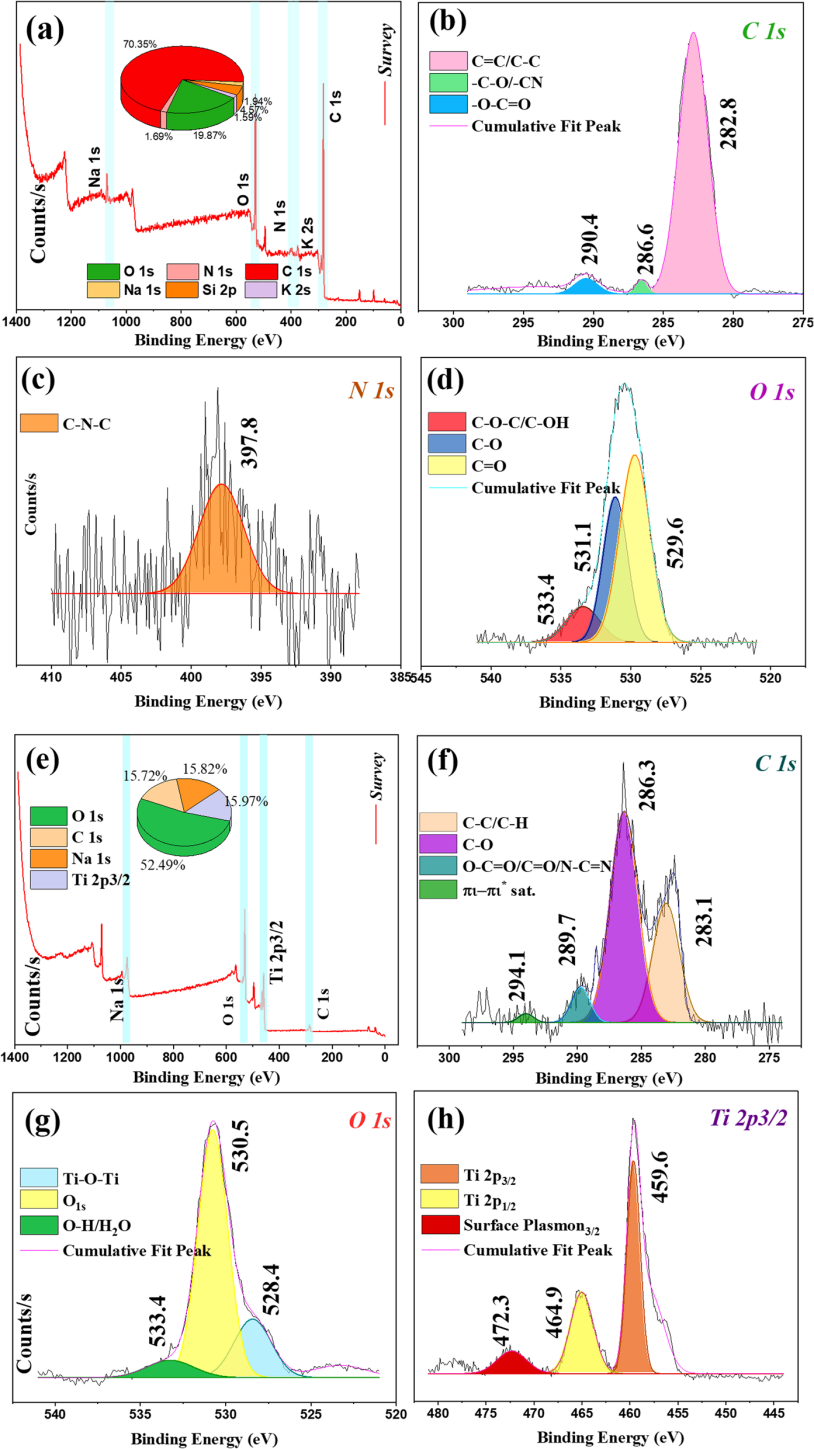
**a** XPS full scan survey spectrum of synthesized C-CQDs, deconvoluted **b** C 1*s*, **c** N 1*s*, and **d** O 1*s* high-resolution XPS spectra of synthesized C-CQDs; **e** XPS full scan survey spectrum of synthesized C-CQDs/TNRs, deconvoluted **f** C 1*s*, **g** O 1*s*, and **h** Ti 2*p*_3/2_ high-resolution XPS spectra of synthesized C-CQDs/TNRs

The O 1*s* high-resolution spectrum (Fig. 6b) displayed two components at 529.9 and 531.1 eV, which were attributed to Ti–O bonds and metal carbonate/C=O species (Yuan et al., 2025). The Ti 2*p* deconvolution (Fig. 6c) exhibited two peaks at 459.2 and 465.07 eV, corresponding to Ti 2*p*_3/2_ and Ti 2*p*_1/2_ orbitals of Ti^4^⁺ (Huang et al., 2025). As can be seen in Fig. 7a, elements such as C, O, N, Na, Si, and K were detected with binding energies of approximately 282.0, 530.0, 397.0, 1071.0, 99.0, and 379.0 eV, corresponding to C 1*s* (70.4%), O 1*s* (19.9%), N 1*s* (1.7%), Na 1*s* (1.9%), Si 2*p* (4.6%), and K 2*s* (1.6%).

As depicted C 1*s* deconvolution spectrum (Fig. 7b) exhibits three notable peaks at 282.8, 286.6 and 290.4 which were assigned to –C=C/–C–C, –C–N/–C=O, and –O–C=O functionalities, respectively (Kaur et al., 2024). The O 1*s* spectrum (Fig. 7c) revealed peaks at 529.6, 531.1, and 533.4 eV, corresponding to C=O, C–O, and C–O–C/C–OH groups (Kundu et al., 2024; Wang et al., 2022). Additionally, the N 1*s* spectrum (Fig. 7d) exhibited a peak at 397.8 eV, indicative of *sp*^2^-hybridized nitrogen in pyridine-like configurations (Garg et al., 2025; Krishnaiah et al., 2025). Figure 7e presents the XPS spectra for the C-CQDs/TNRs nanopowders, highlighting prominent peaks associated with O (52.5%), C (15.7%), Na (15.8%), and Ti (16.0%) located at binding energies of 530.0, 286.0, 1071.0, and 459.0 eV, respectively. Deconvolution of the C 1*s* peak (Fig. 7f revealed contributions from C–C/C–H (283.1 eV), C–O (286.3 eV), –O–C=O/C=O/N–C=O (289.7 eV), and π–π* transitions (294.1 eV), which is alignes well with findings reported in the literature (Liyanaarachchi et al., 2023; Lu et al., 2023; Özdemir et al., 2022; Shi et al., 2024). The O 1*s* spectrum of the composite (Fig. 7g) displayed three peaks at 528.4, 530.5, and 533.4 eV, corresponding to Ti–O–Ti, lattice oxygen, and surface hydroxyl groups, respectively (Guo et al., 2024; Vanlalhmingmawia et al., 2025; Wu et al., 2024). Figure 7h illustrated that the characteristic peaks for Ti 2*p*_1/2_ and Ti 2*p*_3/2_ in TNRs are situated at 459.6 and 464.9 (Hu et al., 2024). Moreover, single surface plasmon interactions are evident through the collective oscillations of free electrons at the material's surface. This small peak observed at 472.3 eV is linked to surface plasmons corresponding to Ti 2*p*_3/2_ (Jaeger & Patscheider, 2012). Compared to pristine TNRs, the Ti 2*p3/2* binding energy in the composite increased by 0.2 eV, while Ti 2*p*_1/2_ decreased by 0.4 eV, confirming successful integration of C-CQDs onto the TNRs surface. This electronic interaction facilitates electron transfer from the carbon dots to the titanium atoms, enhancing the photocatalytic activity of the hybrid nanocomposite.

### Photocatalytic performance C-CQDs/TNRs

The photocatalytic performance of C-CQDs/TNRs nanocomposites synthesized by varying the volume fraction of the C-CQDs solution (1, 5, 10, 15, 20, and 25 vol%, corresponding to 0.1–2.5 mL in a total volume of 10 mL) was investigated against pollutants including methylene blue (MB), hexavalent chromium (Cr(VI)), *E. coli*, and *A. niger*. Since the catalytic behavior of pristine TNRs toward MB, Cr(VI), and microbial species has been extensively documented in previous work (Koç Keşir & Yılmaz, 2024), their performance was not reassessed in this study. Among all prepared composites, C-CQDs@25/TNRs demonstrated the highest photocatalytic activity for the degradation of MB and Cr(VI). Consequently, this particular nanocomposite was selected for detailed evaluation, including in-depth photo-inactivation experiments and comprehensive structural and morphological characterization.

**Fig. 8:**
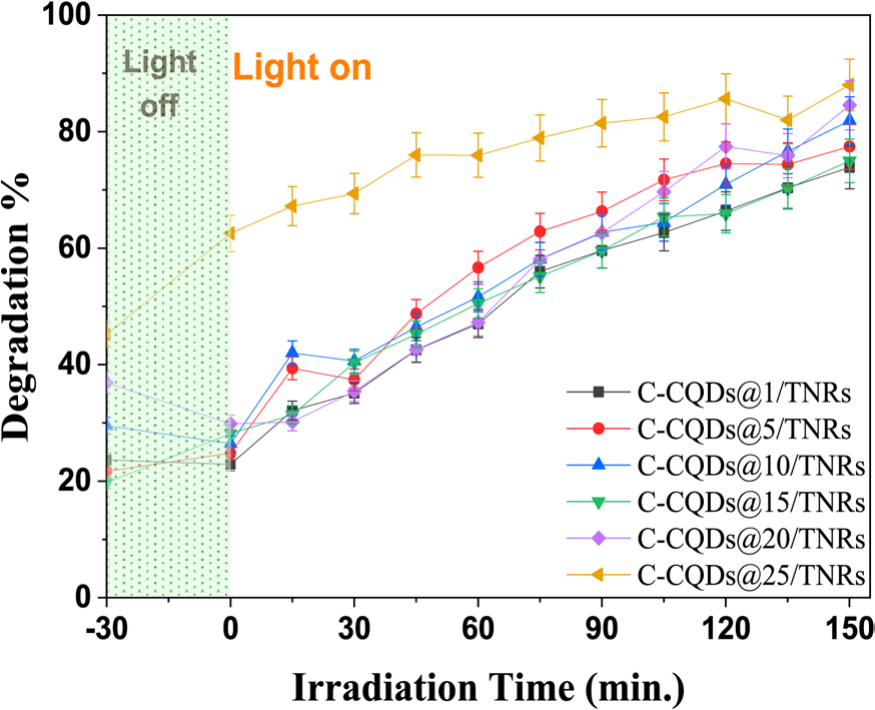
Photocatalytic degradation rate of MB in the presence C-CQDs/TNRs under UV-A ([MB]_0_: 10 ppm, catalyst mass: 0,1 g/L, λ: 365 nm, t:150 min., X:1–25 mL, volume percentage (vol%) of C-CQDs in the total suspension)

As illustrated in Fig. 8, the C-CQDs/TNRs nanocomposites exhibited significantly enhanced photocatalytic activity compared to pristine TNRs. In particular, the C-CQDs@25/TNRs composite achieved an MB degradation efficiency of 88.04% within 150 min, whereas pristine TNRs reached only 60.3%. Control experiments conducted in the dark confirmed that adsorption contributed only 32.9% of the total removal and rapidly reached equilibrium. In contrast, under light irradiation, a continuous decrease in MB concentration was observed, indicating that photocatalytic degradation rather than adsorption dominated the removal process. The enhanced photocatalytic performance of C-CQDs/TNRs is attributed to the improved charge separation and extended light absorption induced by C-CQDs, which promote reactive oxygen species generation. This demonstrates the substantial potential of the hybrid nanocomposite for wastewater treatment applications. The photocatalytic performance of the composites followed the order: NS-CQDs (25)/TNRs (88.03%) > NS-CQDs (20)/TNRs (84.53%) > NS-CQDs (10)/TNRs (81.89%) > NS-CQDs (5)/TNRs (77.43%) > NS-CQDs (15)/TNRs (74.98%) > NS-CQDs (1)/TNRs (73.87%). Increasing the C-CQDs content beyond 25–30% yielded only a marginal improvement of 2.1% in removal efficiency, indicating an optimal loading of 25% for maximum performance. These findings confirm that integrating C-CQDs into the TNRs framework markedly enhances photocatalytic efficiency under visible-light irradiation. The improvement can be attributed to modifications in the optical properties of the composite. Diffuse reflectance spectroscopy (DRS) results revealed that the incorporation of C-CQDs narrows the bandgap of TNRs, enabling stronger absorption of both UV and visible light and facilitating photocatalytic activity at longer wavelengths (lower energy levels) (Meena et al., 2024a, 2024b).

**Fig. 9:**
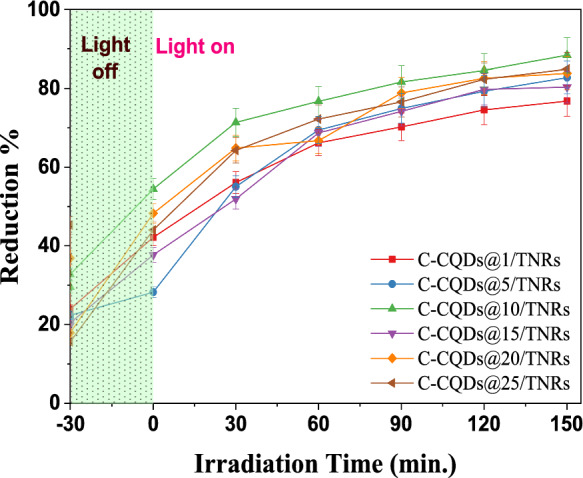
Photocatalytic degradation rate of Cr (VI) in the presence C-CQDs/TNRs NRs under UV-A ([Cr(VI)]_0_: 10 ppm, catalyst mass: 50 mg (1 g/L), pH:1.5, [EDTA]_0_: 10 mm, V_EDTA_: 50 μL, λ: 365 nm, t:150 min., X:1–25 mL, the volume percentage (vol%) of C-CQDs in the total suspension)

Figure 9 depicts the temporal evolution of Cr(VI) removal using C-CQDs/TNRs composites compared to pristine TNRs. The photocatalytic efficiencies of the various nanocomposites follow the sequence: C-CQDs (10)/TNRs (88.39%) > C-CQDs (25)/TNRs (84.87%) > C-CQDs (20)/TNRs (83.82%) > C-CQDs (5)/TNRs (82.75%) > C-CQDs (15)/TNRs (80.32%) > C-CQDs (1)/TNRs (76.76%). For comparison, photolysis alone achieved only 7.56% Cr(VI) removal over 150 min, whereas pristine TNRs reached 51.46% under the same conditions (Koç Keşir & Yılmaz, 2024). The results demonstrate that the introduction of C-CQDs significantly enhances Cr(VI) removal, with efficiencies reaching up to 88.36% as the C-CQDs content increases. The substantial decrease in Cr(VI) concentration suggests an effective photocatalytic reduction process, which is commonly attributed to the conversion of Cr(VI) to the less toxic Cr(III) species under photocatalytic conditions. After 150 min of UV-A irradiation, less than 12% of the initial Cr(VI) remained in solution. According to the Pourbaix diagram for Cr(VI), the predominant species in aqueous solutions at pH < 6.5 are H_2_CrO₄(aq) and HCrO₄⁻, while CrO_4_^2−^ becomes dominant at pH > 6.5. In our study, the point of zero charge (pH_p_zc) for C-CQDs/TNRs was determined to be approximately 6.24 in a 10⁻^2^ M KCl solution over a pH range of 3–11. When the solution pH is below the pH_p_zc, the surface of C-CQDs/TNRs becomes protonated, promoting stronger electrostatic attraction between the positively charged catalyst surface and negatively charged Cr(VI) species, thereby facilitating enhanced Cr(VI) removal (Chang et al., 2022; Wei et al., 2023). The interaction of photogenerated electrons with hexavalent chromium under acidic conditions, where Cr(VI) predominantly exists as Cr_2_O_7_^2−^, can be expressed as follows:

5 $$ {\mathrm{Cr}}_{2} {\mathrm{O}}_{7}^{2 - } + 14{\mathrm{H}}^{ + } + 6e^{ - } \to 2{\mathrm{Cr}}^{3 + } + 7{\mathrm{H}}_{2} {\mathrm{O}} $$

Accordingly, all Cr(VI) photo-reduction experiments were performed at pH 1.5 to ensure optimal removal efficiency. It should be noted that the initial removal observed during the dark period is primarily attributed to adsorption of MB and Cr(VI) onto the catalyst surface. Upon light irradiation, a distinct increase in removal efficiency is observed, indicating that the subsequent pollutant removal is predominantly governed by photocatalytic degradation and reduction processes rather than adsorption alone.

To gain insight into the photocatalytic degradation pathway of MB and to determine the predominant reactive species involved in the C-CQDs/TNRs system, a series of reactive species scavenging experiments was conducted. Specific quenchers, namely isopropyl alcohol (IPA, 0.1 mol L^−1^), benzoquinone (BQ, 0.1 mol L^−1^), and ethylenediaminetetraacetic acid disodium salt (EDTA-2Na, 0.1 mol L^−1^), were employed to selectively inhibit ·OH radicals, ·O_2_^–^ radicals, and photogenerated holes (h⁺), respectively.

**Fig. 10:**
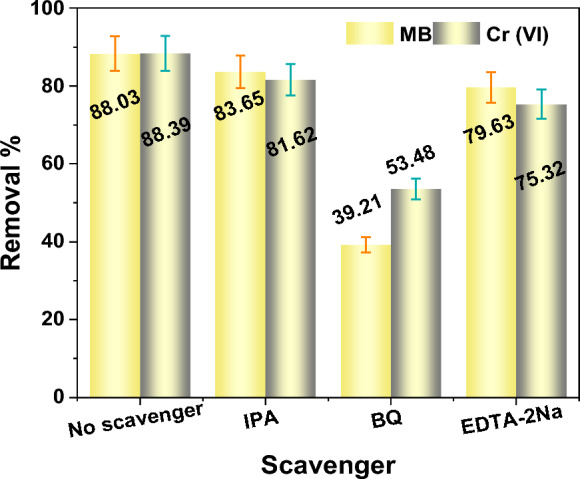
Effect of different scavengers on the MB degradation by C-CQDs/TNRs nanoparticles

As illustrated in Fig. 10, the presence of EDTA and IPA caused only negligible changes in the removal efficiencies of Cr(VI) and MB, indicating that photogenerated holes (h⁺) and hydroxyl radicals (·OH) do not play dominant roles in the removal processes. In contrast, the addition of benzoquinone (BQ) resulted in pronounced suppression, with the removal efficiencies decreasing to 53.48% for Cr(VI) and 76.63% for MB, respectively. These results clearly demonstrate that superoxide radicals (·O_2_^–^) are the primary reactive species responsible for the reduction of Cr(VI) and the degradation of MB in the system. The observed enhancement in photocatalytic performance and dominant superoxide-mediated reaction pathway highlight that the choice of *Carduus *spp.–derived CQDs contributes not only as a sustainable carbon source but also as an active electronic modulator in the CQDs/TiO_2_ system, distinguishing this work from previously reported biomass-based CQD photocatalysts.

Table 1 presents pertinent data from the photocatalytic performance of CQD-decorated nanomaterials made from various biomass, which were used to compare the results of our work with similar studies. Among the various biomass-derived composites in earlier research, created C-CQDs/TNRs shown a notable removal efficiency rate when irradiation period, material amount, and light source power were taken into consideration. In general, it is also important to note that photocatalytic research uses light sources with high power in the literature. As a result, nutshell-derived C-CQD/TNRs exhibit higher photocatalytic capabilities than CQDs/TiO_2_ made with different green precursors.

**Table 1:** Comparison of photocatalytic performances for methylene blue (MB) and Cr (VI) removal efficiency with CQDs modified nanomaterials obtained disparate biomass

Biomass	Synthesis route	Model pollutant	Reaction conditions	Degradation efficiency	References
	Hydrothermal	1.5 × 10^−5^ M MB	3 mg/L CQD 20 W UV lamp Irradiation time: 40 min	100%	Najjar et al. (2022)
	–	10 ppm MB	NCQDs/TiO_2_/PVA film Xenon lamp (1000 W/m^2^) Irradiation time: 180 min	98.2%	Waluyo et al. (2024)
	Solvothermal-pyrolysis	60 ppm MB	1.0 g/L catalyst 300 W Hg (20 min) and 350 W Xe lamps (240 min)	100%	Gao et al. (2024)
	Hydrothermal	10 ppm	0.1 g/L NCQDs/TiO_2_ 300-W Xenon lamp Irradiation time: 60 min	93.1%	Jin et al. (2023)
	Hydrothermal	1 ×10 ^−5^ M MB	1 g/L RGO/CDs 500 W halogen lamp Irradiation time: 140 min	87%	Shahraki et al. (2023)
	Thermally pyrolyzed	5 ppm MB	0.1 g/L mg C-dot 300 W UV bulb Irradiation time: 90 min	94.8%	Kumar and Kumar (2024)
	Hydrothermal	10 ppm Cr (VI)	2.0 g/L of P-CDs/TiO_2_@CS simulating sunlight (300 W) irradiation time: 60 min	99.64%	Ma et al. (2025))
	Hydrothermal	10 µg/L MB	1 g/L NCQDs/TiO_2_ 80 W LED lamp Irradiation time: 240 min	86.16%	Heng et al. (2022)
	Hydrothermal	5–50 ppm MB	0.6 g/L WO_3_/N-CQDs 80 W halogen lamp (X2) Irradiation time: 240 min	96.86–92.93%	Nugraha et al. (2021)
	Hydrothermal	–	1.0 g/L CQDs/CeO_2_ 380W Xe lamp Irradiation time: 60 min	80%	Gong et al. (2019)
	Hydrothermal	25 ppm MB	1.25 g/L ZnO/CQDs Tunsten lamp Irradiation time: 150 min	68%	Parveen et al. (2025)
	Hydrothermal	10 ppm Cr (VI)	1.0 g/L CQDs/g-C_3_N_4_ 300 W Xe lamp Irradiation time: 20 min	99.5%	Xu et al. (2025b)
	Hydrothermal	20 ppm Cr (VI)	0.5 g/L Bi_7_O_9_I_3_/g-C_3_N_4_ 300 W Xe lamp Irradiation time: 60 min	100%	Zhu et al. (2023)
	Hydrothermal	50 ppm Cr (VI)	2.0 g/L of the RN-CDs/TiO_2_ 300 W xenon lamp Irradiation time: 60 min	98.61%	Ma et al. (2024)
	Pyrolysis	10 ppm MB and Cr (VI)	0.1 g/L C-CQDs/TNRs 6W (X2) dual lamp (light intensity 350 mW/cm^2^) Irradiation time: 150 min	88.03% for MB 88.39% for Cr (VI)	This work

**Fig. 11:**
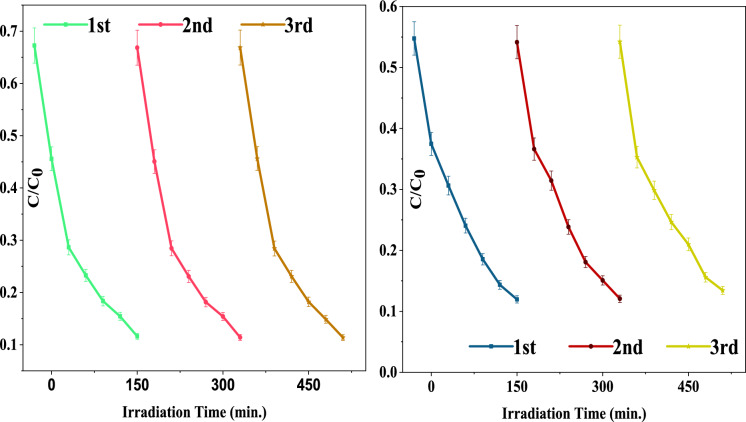
Reusability of C-CQDs/TNRs composites for Cr (VI) (left) and MB (right)

The reusability of the C-CQDs/TNRs nanocomposites was examined over three consecutive cycles of MB and Cr(VI) degradation under identical conditions. As shown in Fig. 11, the composites retained their photocatalytic activity without any observable decline, highlighting their remarkable photo-stability and recyclability, which is essential for practical wastewater treatment applications.

**Fig. 12:**
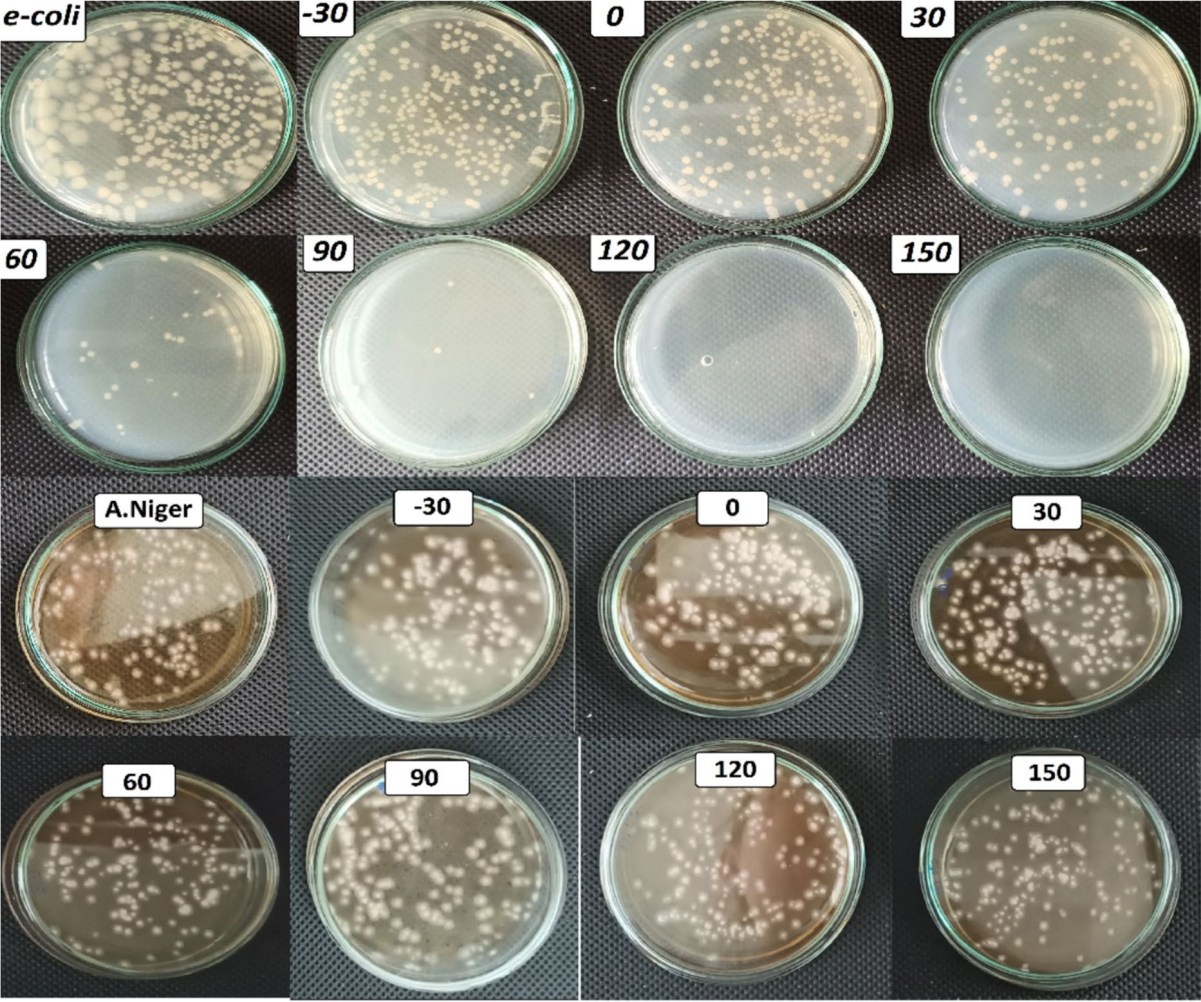
Inactivation performance of C-CQDs/TNRs composites against *E. coli* and *A. niger*

The antimicrobial properties of C-CQDs/TNRs were further assessed against Gram-negative bacteria (*E. coli*) and fungi (*A. niger*) under visible-light irradiation (Fig. 12).

Consistent with previous reports (Koç Keşir & Yılmaz, 2024), pristine TNRs exhibited limited photo-inactivation. For *A. niger*, no significant reduction in fungal density was observed over 150 min. In contrast, *E. coli* demonstrated pronounced susceptibility: initially intact at 0 min, bacterial cells progressively deteriorated with prolonged exposure, ultimately resulting in complete cell destruction. The improved antibacterial activity observed for C-CQDs/TNRs may be associated with enhanced photo induced charge separation and increased generation of reactive species, which are commonly reported for CQD-modified semiconductor systems. However, it should be noted that parameters such as bandgap narrowing, charge carrier mobility, and reactive oxygen species generation were not directly quantified in this study. Therefore, these effects are discussed as plausible contributing factors based on literature reports rather than definitive mechanisms (Mahmudin et al., 2024; Singh et al., 2025). In contrast, the antifungal activity against *Aspergillus niger* showed no statistically significant variation between pristine TNRs and C-CQDs/TNRs samples. This observation can be attributed to the intrinsic resistance of fungal cells to UV radiation and oxidative stress, as well as possible electrostatic interactions between negatively charged surface functional groups on the nanocomposites (identified by FTIR analysis, Fig. 3) and the negatively charged fungal cell walls. As a result, the antimicrobial effect of the C-CQDs/TNRs composites appears to be microorganism-dependent (Zhang et al., 2021c; Zhao et al., 2021). Although UV irradiation alone can contribute to microbial inactivation, the enhanced antibacterial performance observed in the presence of the photocatalyst indicates an additional photocatalytic effect. This enhancement is attributed to the generation of reactive oxygen species under irradiation, which act synergistically with UV exposure to damage microbial cell structures.

### Cytotoxicity assay

**Fig. 13:**
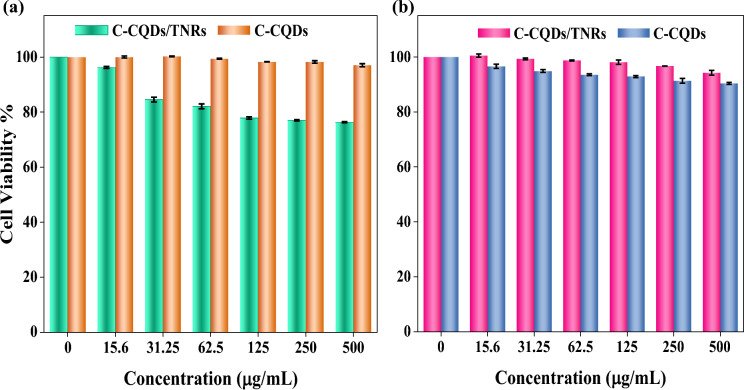
Relative viabilities of the ARPE-19 cells (**a**) and MCF-7 (**b**) treated with C-CQDs and C-CQDs/TNRs at different concentrations for 48 h

In vitro cytotoxicity assays were carried out using ARPE-19 retinal epithelial cells and MCF-7 carcinoma cells at concentrations up to 500 μg mL⁻^1^ for 48 h, and the corresponding results are presented in Fig. 13. According to the MTT assay results, neither C-CQDs nor C-CQDs/TNRs composites induced significant inhibition of cellular viability over the investigated concentration range.

For ARPE-19 cells, pure C-CQDs exhibited excellent cytocompatibility, maintaining cell viability close to 100% even at the highest tested concentration. In contrast, C-CQDs/TNRs composites showed a slight concentration-dependent decrease in viability; however, cell viability remained above ~ 75% at all concentrations. Since materials maintaining cell viability above 70% are generally considered non-cytotoxic according to ISO 10993-5 guidelines, the composites can be regarded as biocompatible toward normal retinal cells. In the case of MCF-7 carcinoma cells, both C-CQDs and C-CQDs/TNRs demonstrated consistently high cell viability (> 85%) across all concentrations, indicating negligible cytotoxic effects. The relatively higher tolerance of MCF-7 cells may be attributed to their altered metabolic activity and stress-response mechanisms compared to non-cancerous cells. The low cytotoxicity observed for the C-CQDs/TNRs nanocomposites can be associated with the biomass-derived nature of the C-CQDs and the presence of oxygen-containing surface functional groups, which enhance hydrophilicity and mitigate adverse cell–material interactions. Additionally, the CQDs layer may act as a surface passivation coating on TNRs, reducing direct contact between cells and potentially reactive metal oxide surfaces. It should be noted that the MTT assay reflects short-term metabolic activity and does not fully represent long-term or genotoxic effects. Therefore, while the present results confirm good cytocompatibility after 48 h of exposure, further long-term and complementary biological assays are required for comprehensive biosafety evaluation.

## Conclusions

In summary, plant-derived carbon quantum dots (C-CQDs) and TiO_2_ nanorods (TNRs) were synthesized via a fixed-bed pyrolysis reactor and hydrothermal methods, respectively, and combined through a wet impregnation process to form hybrid composites. Under UV-A irradiation, the C-CQDs/TNRs composites exhibited improved removal performance for organic dye (MB) and Cr(VI) compared to pristine TNRs. The observed removal efficiencies result from the combined contributions of adsorption and irradiation-assisted processes, with adsorption playing a notable role, particularly during the initial stages. XPS and EDS analyses indicated increased oxygen content on the hybrid surface, while FTIR spectroscopy suggested the formation of Ti–O–C linkages between C-CQDs and TNRs. Among the investigated samples, C-CQDs@25/TNRs showed the highest removal efficiencies, achieving 88.03% for MB and 88.39% for Cr(VI), exceeding those of unmodified TNRs. Antimicrobial experiments revealed enhanced bacterial inactivation under UV-A irradiation in the presence of C-CQDs/TNRs compared to pristine TNRs; however, UV-A exposure itself contributed significantly to microbial inhibition. Limited antifungal activity against *Aspergillus niger* was observed, highlighting the microorganism-dependent nature of the system. Reusability tests demonstrated that the composites retained their removal performance over three successive cycles. Furthermore, in vitro cytotoxicity assays confirmed that both C-CQDs and C-CQDs/TNRs maintained acceptable cytocompatibility toward human cell lines within the tested concentration range. Overall, this study demonstrates that biomass-derived C-CQDs can be effectively integrated with TiO_2_ nanorods to produce multifunctional composites for environmental remediation under irradiation conditions. Further investigations involving detailed mechanistic studies and comprehensive biological assessments are required to fully elucidate the individual contributions of adsorption, irradiation, and photo-induced processes.

## Data Availability

No datasets were generated or analysed during the current study.
